# Supplementary material for: A New Method for Rapid Subcellular Localization and Gene Function Analysis in Cotton Based on Barley Stripe Mosaic Virus
Source: Plants (Basel). 2022 Jul 1;11(13):1765. doi: 10.3390/plants11131765 (PMC9268801; doi:10.3390/plants11131765)
Supplement: Supplementary file 1 [file plants-11-01765-s001.zip › plants-1758650-supplementary.pdf]

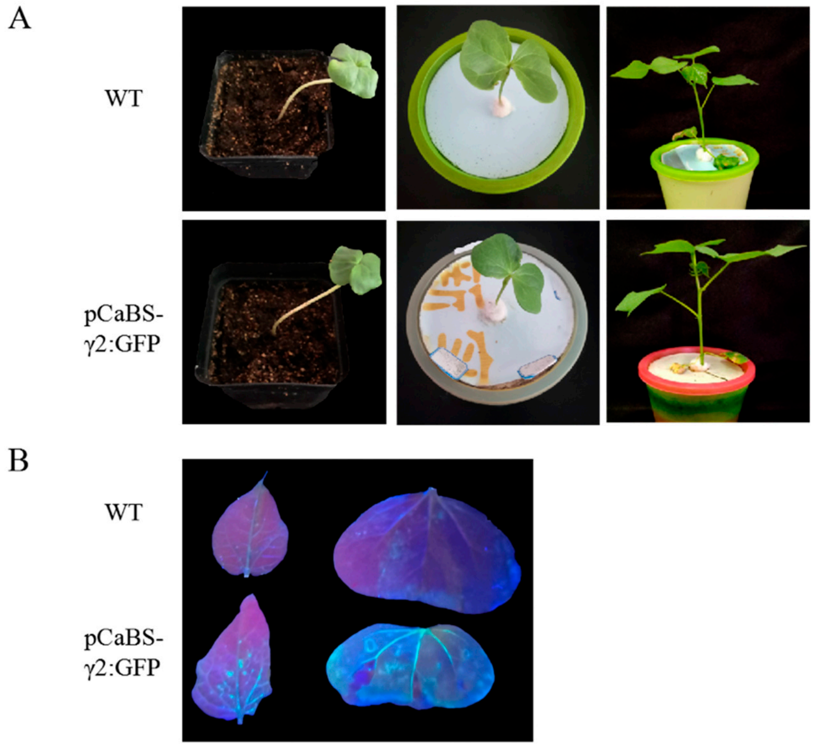

Figure S1. Seed imbibition method does not affect cotton growth and can stably express gene over a long period of time. A. The growth of cotton under different treatment methods, from left to right, are soil culture stage, hydroponic stage, and 4-6 true leaf stage; B. Fluorescence of real leaves (left) and cotyledons (right) of cotton in the true leaf stage under ultraviolet light.

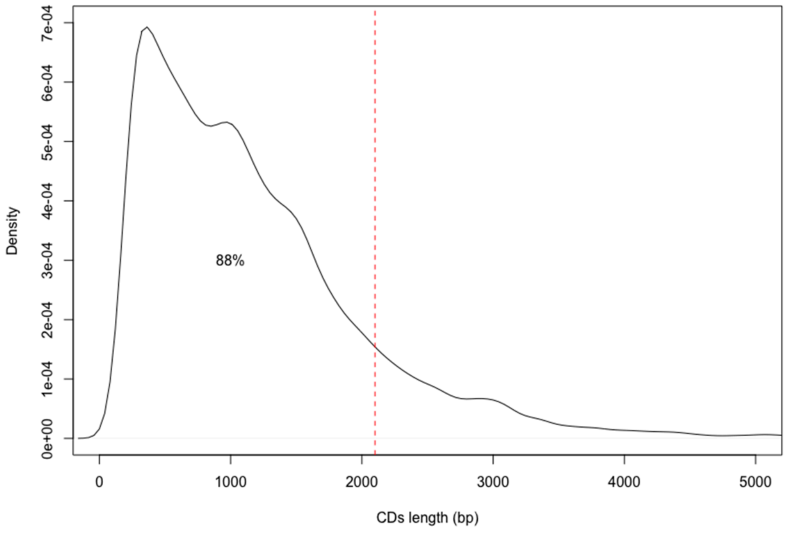

Figure S2. Gene length density of cotton.

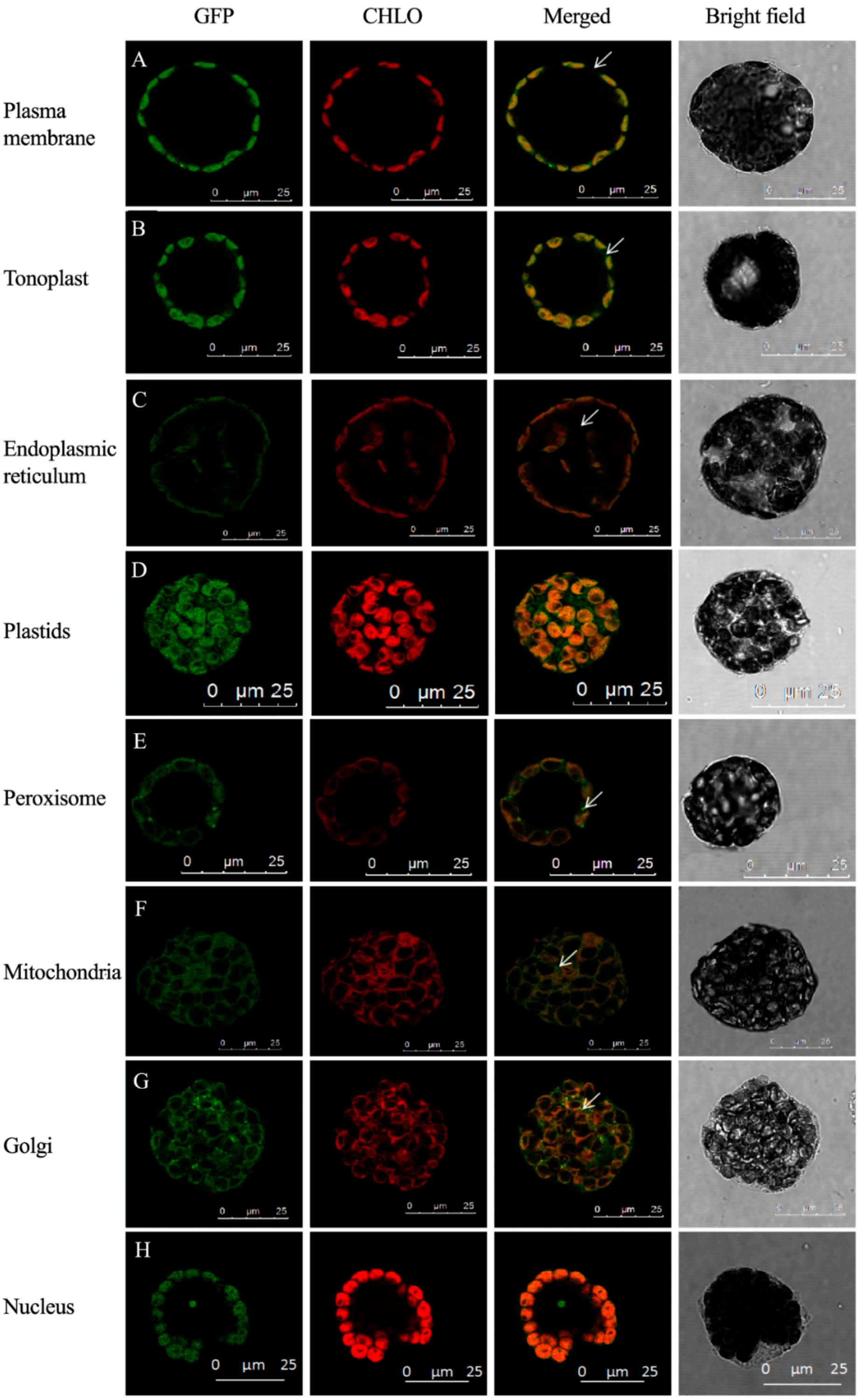

Figure S3. Ti vector transient transformation-mediated subcellular localization of GFP fusion-marker genes targeting in cotton protoplast. The localization constructs were transiently expressed in cotton protoplasts by PEG-mediated transformation and observed by confocal microscopy. A. The green fluorescence of *GhPIP2:GFP* was expressed transiently in the plasma membrane; B. The tonoplast marker *GhTIP2:GFP* was found in the membrane protruding into the interior of the cell; C. Fluorescence of endoplasmic reticulum marker *GhSPP:GFP* in cotton protoplasts revealed the typical network morphology of the ER; D. The plastid marker *GhClpD:GFP* in cotton protoplasts showed GFP fluorescence completely overlapped with chloroplast auto-fluorescence; E. The fluorescence of the peroxisomal marker *GhAPX3:GFP* showed as spherical spots localized near chloroplasts; F. The typical granular, thread and randomly distributed mitochondria were observed in leaf protoplasts about marker *GhALDH2:GFP*; G.

The fluorescence of the Golgi marker *GhMNS1:GFP* showed a punctate staining pattern smaller than 1 μm ; H. The green fluorescence of the final marker *GhTAF2:GFP* was well targeted to the nucleus of the protoplasts.

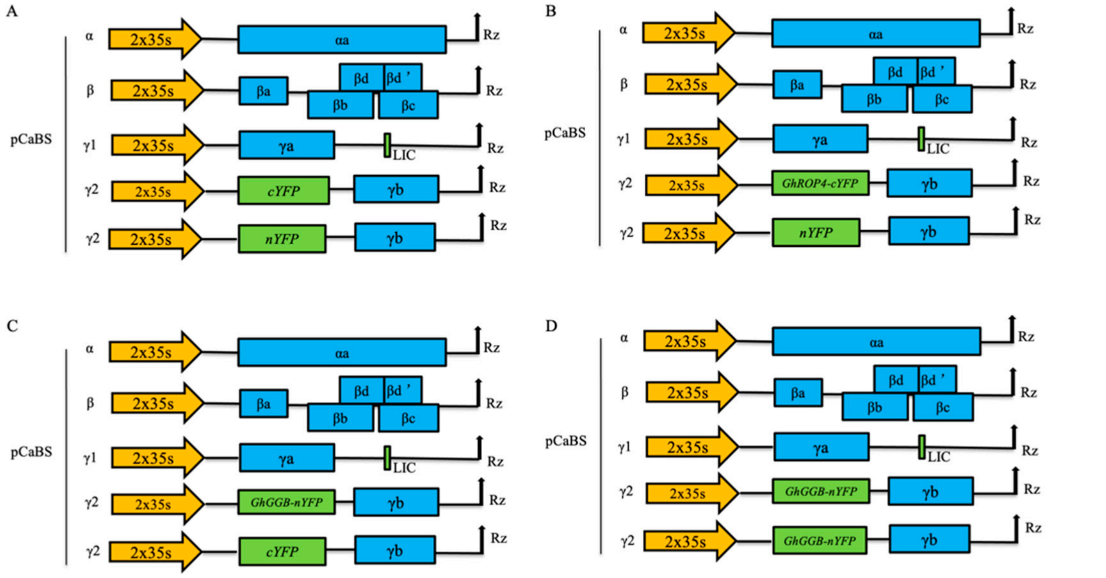

Figure S4. Schematic representation of BSMV recombinant constructs used for BiFC assay. A. The pCaBS-γ2:*nYFP* (amino acids 1 to 156; nYFP) + pCaBS-γ2:*cYFP* (amino acids 157 to 239; cYFP); B. pCaBS-γ2:*GhROP4-cYFP* + pCaBS-γ2:*nYFP*; C. pCaBS-γ2:*GhGGB-nYFP* and pCaBS-γ2:*cYFP*; D. pCaBS-γ2:*GhROP4-cYFP* + pCaBS-γ2:*GhGGB-nYFP*.

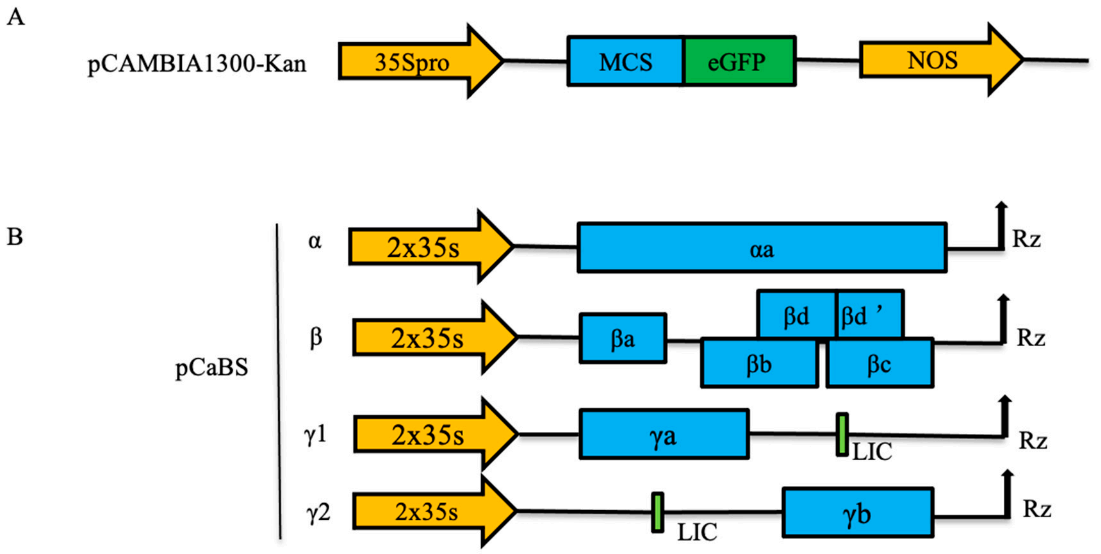

Figure S5. Schematic representation of the expression construct and virus vector. A. Schematic representation of the expression vector; B. Schematic representation of the four-component BSMV system. In the pCaBS-α, pCaBS-β, pCaBS-γ1, and pCaBS-γ2 vectors, the α, β, γ1, and γ2 cDNAs were cloned between the double cauliflower mosaic virus 35S promoter and a ribozyme sequence (Rz) in the pCass4-Rz plasmid. A LIC cloning site containing an *ApaI* site was inserted into the γ2 genome to substitute the γb genes from the original γ genome.

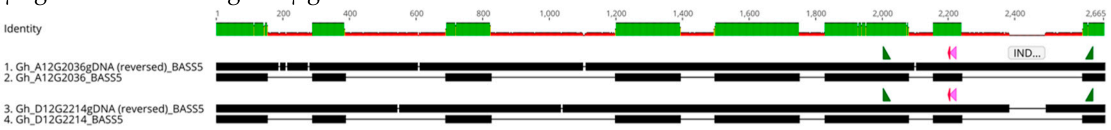

Figure S6. The alignment of cDNAs and genome DNA sequence between *GhBASS5A* and *GhBASS5D*. The black boxes and lines indicated exons and introns, respectively; the two green triangles showed the

position of primers for CAPs analysis; the purple arrows indicated the PAM site; the gray INDEL box represented the 109 bp indel sequence between *gGhBASS5A* and *gGhBASS5D*.

Table S1. Cotton organelle marker genes and their primer information (sequence underlined is the position of the introduced cleavage site).

Organelles	Cotton label	Primer sequence(5′ to 3′)	Length	Temperature
Nucleus (NU)	Gh_A07G0281	F:CGAGGATCCATGAACCACAACCCGCAATCC<br>R:GCATCTAGAATTCCTCTCTAGAAGCGGGATCG	411 bp	62 °C
Endoplasmic reticulum (ER)	Gh_A05G0593	F:CGCGGATCCATGAAGAACACTGAAAGACTCGCC ʻ<br>R:TGCTCTAGATACATCAAATCTCAATGCTAGGGC'	765 bp	62 °C
Plasma membrane (PM)	Gh_D03G1822	F:CGAGGATCCATGACTAAGGATATTGAGACCACGG<br>R:GCATCTAGAAAGCATTGCTCCTGAAAGATCCAAGG	876 bp	61 °C
Mitochondria (MT)	Gh_A12G2471	F:CGAGGATCCATGGCAGCTCGTAGAATCTCTTC<br>R:GCATCTAGACAACCATGCTGGATTCTTCAAAGG	1623 bp	62 °C
Tonoplast (TP)	Gh_A04G1393	F:CGATGGATCCATGCCGATCAGAAACATAGCAG<br>R:GCATCTAGAAATAATCGGTGGTTGGGAGCTGCTCG	756 bp	62 °C
Plastids (PL)	Gh_A09G2205	F:CGCGGATCCATGGAGGTTTTATCTTCTTCGTCTTC<br>R:TGCTCTAGATGTACCAGATCCTATAAGTGTGTGG	1200 bp	64 °C
Golgi body (GB)	Gh_A02G0907	F:CGCGGATCCATGGCGAGGAGTAGATCATCGTCAT<br>R:TGCTCTAGACAGTAGTAGTATCCCAAGCAGGTAG	628 bp	62 °C
Peroxisome (PR)	Gh_A03G1812	F:CGAGGATCCATGGCGTTTCCAGTAGTCGATACCG<br>R:GCATCTAGAACTTCATTCTTTTGCGGACCTCGT	867 bp	63 °C

Table S2. LIC primers and qPCR primers used in this study

Primer	Primer sequence(5′ to 3′)	Length
LIC-NU-GFP	F:AAGGAAGTTTAAATGAACCACAACCCGCAAT<br>R:CGGGCCAGCCACCGCCACCAGTTTACTTGTACAGCTCGTCCATGC	1137 bp
LIC-MT-GFP	F:AAGGAAGTTTAAATGGCAGCTCGTAGAATC<br>R:CGGGCCAGCCACCGCCACCAGTTTACTTGTACAGCTCGTCCATGC	2340 bp
LIC-ER-GFP	F:AAGGAAGTTTAAATGAAGAACACTGAAAGAC<br>R:CGGGCCAGCCACCGCCACCAGTTTACTTGTACAGCTCGTCCATGC	1491 bp
LIC-PM-GFP	F:AAGGAAGTTTAAATGGAGGGTAAAGAAGAAG<br>R:CGGGCCAGCCACCGCCACCAGTTTACTTGTACAGCTCGTCCATGC	1593 bp
LIC-TP-GFP	F:AAGGAAGTTTAAATGCCGATCAGAAACATAG<br>R:CGGGCCAGCCACCGCCACCAGTTTACTTGTACAGCTCGTCCATGC	1482 bp
LIC-PL-GFP	F:AAGGAAGTTTAAATGGAGGTTTTATCTTCT<br>R:CGGGCCAGCCACCGCCACCAGTTTACTTGTACAGCTCGTCCATGC	1926 bp
LIC-GB-GFP	F:AAGGAAGTTTAAATGGCGAGGAGTAGATCAT<br>R:CGGGCCAGCCACCGCCACCAGTTTACTTGTACAGCTCGTCCATGC	1354 bp
LIC-PR-GFP	F:AAGGAAGTTTAAATGGCGTTTCCAGTAGTC<br>R:CGGGCCAGCCACCGCCACCAGTTTACTTGTACAGCTCGTCCATGC	1593 bp
LIC-GFP	F:AAGGAAGTTTAAATGGTGAGCAAGGGCGAG<br>R:CGGGCCAGCCACCGCCACCAGTTTACTTGTACAGCTCGTCCATGC	720 bp
LIC-GhBASS5-GFP	F:AAGGAAGTTTAAATGAGTTCAACCACTGG<br>R:CGGGCCAGCCACCGCCACCAGTTTACTTGTACAGCTCG	1950 bp
qPCR-GFP	F:GGTGATGTTAATGGGCAC<br>R:TCAGGCATGGCACTCTTGA	212 bp
UBQ7	F:AGAGGTCGAGTCTTCGGACA<br>R:GCTTGATCTTCTTGGGCTTG	146 bp
A-GFP	F:ATGGTGAGCAAGGGCGAG<br>R:TTACTTGTACAGCTCGTCCATGC	720 bp

Table S3. Primers used for detection of gene editing

Primer	Primer sequence(5′ to 3′)	Purpose of primers
GhBASS5-BamHI	F:GTTATTTCCTCAGTGGTTCACT R:AGCGAAGCCCATCAATGACA	Amplification of DNA fragment
BSMV-α	F:CCATCGTCGATTCCGTGGAT R:CCTCGCATTTGCATCAGCTC	RT-PCR
BSMV-β	F:GCACCCTAGAACGTGAACGA R:AGCGAAGCAGTCTCTTGTCC	RT-PCR
BSMV-γ1	F:TTCGACTTCAAGTACCCCGC R:TAGTCGAATCAGTAGCAACC	RT-PCR
Cas9N	F:GCTCTAGAATGGATTACAAGGACCACGAC R:CGAGCTCGCTCACCTGAGCCTTCTGGA	RT-PCR
Cas9C	F:GCTCTAGAATGGGCCAGGGGGACTCGCTG R:CGAGCTCTCACTTCTTCTTCTTCGCCTGC	RT-PCR
γ2-Cas9N	F:AAGGAAGTTTAAATGGATTACAAGGACCACGAC R:CGGGCCAGCCACCGCCACCAGTTCAGCTCACCTGAGCCTTCTGGA	Clone γ2-Cas9N
γ2-Cas9C	F:AAGGAAGTTTAAATGGGCCAGGGGGACTCGCTG R:CGGGCCAGCCACCGCCACCAGTTCACTTCTTCTTCTTCGCCTGCC	Clone γ2-Cas9C

Video S1. Time series of fluorescence signal of GhBASS5-GFP in cotton leaf.
